# Does rheumatic fever-induced myocardial involvement improve? Three-dimensional echocardiography and the answer to this question

**DOI:** 10.22088/cjim.15.3.557

**Published:** 2024-08-01

**Authors:** Zahra Jabbary

**Keywords:** Rheumatic fever, Myocardial involvement, Three-dimensional echocardiography

**Dear Editor**

Acute rheumatic fever (ARF) is the result of the body’s autoimmune response to a throat infection caused by Streptococcus pyogenes, also known as group A streptococcus (GAS) bacteria. ARF is the main cause of acquired heart disease in children and young adults (1).

The burden of rheumatic fever is uncertain due to its diagnosis complexity. The worldwide incidence rate is 19/100,000 (range, 5-51/100,000), with highest rates (˃10/100,000) in Eastern Europe, the Middle East, Asia, Africa, Australia, New Zealand, and lowest rates (˂10/100,000) in North America and Western Europe. Although similar in occurrence between men and women, due to the risk of disease during pregnancy, the inherent biological factors, GAS exposure through child rearing, and poor access to resources, women are approximately 1.8 times more likely to develop rheumatic heart disease (RHD) (2).

RF is a multifactorial disease following GAS pharyngitis in a susceptible person living in deprived social conditions. Molecular mimicry theory holds that GAS pharyngitis triggers an autoimmune response in susceptible persons by cross reacting with similar epitopes in the heart, joints, brain, and skin (3). Pathologically, the inflammatory process damages the collagen fibrils and connective tissue ground substance (4).

Cross-reactive immune response leads to transient migratory polyarthritis resulting in the formation of immune complexes, results in Sydenham’s chorea as the antibodies bind to basal ganglia and neuronal cells, causes erythema marginatum and subcutaneous nodules in the skin as antibodies bind to keratin, and ultimately leads to inflammation of the heart valves and myocardium. Although the myocardium heals following this inflammation, it can cause permanent damage to the valves, leading to rheumatic heart disease (5). Rheumatic fever is characterized by transient arthritis and harmful carditis. Thus, in 1884, the French physician Ernst-Charles Laśegue said, that “rheumatic fever licks the joints but bites the heart” (4). Long-term damage to the heart valves caused by ARF, which results from a single severe episode or multiple recurrent episodes of the disease, is known as rheumatic heart disease (5). The mitral valve is the most common valve affected by rheumatic heart disease. Approximately 25% of all patients with rheumatic heart disease have isolated mitral stenosis (MS), and approximately 40 % have a combination of MS and mitral regurgitation (MR). Rheumatic mitral valve disease is still a major health problem, especially in developing countries. Echocardiography is the most important investigation in the diagnosis and planning of the management of MS.

The use of cardiac ultrasound is very necessary to understand the normal function of the heart and is very important for pathophysiological diagnosis. The growing availability of three dimensional echocardiography (3DE) has allowed its applications to expand from establishing reference values for chamber size and elucidating ventricular mechanics to assessing severity of valvular disease and playing key roles in interventional procedures. Several important advantages of 3DE are elimination of geometric assumptions, quantification of volumes of complex geometric shapes, visualization of structures from any perspective, evaluation of lesions in simultaneous multi-plane or multi-slice mode, which are not possible with traditional two dimensional echocardiography (2DE). 3DE has shown to be accurate, simple, versatile and reproducible, and compared to 2DE. It has a better outcome prognosis (6). 

In the study, we conducted on patients with rheumatic stenosis of the mitral valve, we achieved important points (7). First, three dimensional echocardiography allowed us to distinguish nodules from calcification, determine the severity and extent of calcification of each scallop, especially the anterior leaflet scallops, and assess commissural fusion. Second, in some patients with severe mitral valve stenosis, calcification, like a ring, covered the entire annulus of the mitral valve.

Third, due to providing en-face view in 3D echocardiography, we observed that the inner wall of the left atrium, especially the inter-atrial septum wall in these patients, is irregular, rough and sandpaper. And most importantly, in some patients, we observed discrete calcification masses on the inner surface of the wall of the left atrium and left ventricle, around the mitral valve, which reminded us of a rock climbing wall. Our observations indicate that unlike the previous theory which believes that, rheumatic fever can lead to permanent valve damage and the myocardium recovers after involvement, in the long term the process that continues in the valves, can also continue in the myocardium, and lead to permanent myocardial damage. 

## References

[1] Sika-Paotonu D, Beaton A, Raghu A, Steer A, Carapetis J, Ferretti JJ, Stevens DL, Fischetti VA (2016). Acute Rheumatic Fever and Rheumatic Heart Disease. Streptococcus pyogenes: Basic Biology to Clinical Manifestations.

[2] Watkins DA, Johnson CO, Colquhoun SM (2017). Global, Regional, and National Burden of Rheumatic Heart Disease, 1990-2015. N Engl J Med.

[3] Bright PD, Mayosi BM, Martin WJ (2016). An immunological perspective on rheumatic heart disease pathogenesis: more questions than answers. Heart.

[4] Bonow RO, Mann DL, Zipes DP, Libby P (2011). Braunwald's Heart Disease: A Textbook of Cardiovascular Medicine. 9th ed. Philadelphia: Elsevier Science.

[5] Carapetis JR, Beaton A, Cunningham MW (2016). Acute rheumatic fever and rheumatic heart disease. Nat Rev Dis Primers.

[6] Wu VC, Takeuchi M (2017). Three-Dimensional Echocardiography: Current Status and Real-Life Applications. Acta Cardiol Sin.

[7] Toufan M, Jabbary Z, Aghdam NK (2022). Two-dimensional-Wilkins and real-time transesophageal three-dimensional scoring systems: Which one is preferred to mitral valve morphological assessment?. Asian Cardiovasc Thorac Ann.

